# Ranging, Activity and Habitat Use by Tigers in the Mangrove Forests of the Sundarban

**DOI:** 10.1371/journal.pone.0152119

**Published:** 2016-04-06

**Authors:** Dipanjan Naha, Yadvendradev V. Jhala, Qamar Qureshi, Manjari Roy, Kalyansundaram Sankar, Rajesh Gopal

**Affiliations:** 1 Department of Animal Ecology and Conservation Biology, Wildlife Institute of India, Chandrabani, Dehradun, Uttarakhand, India; 2 Department of Landscape level planning and management, Wildlife Institute of India, Chandrabani, Dehradun, Uttarakhand, India; 3 Department of Habitat Ecology, Wildlife Institute of India, Chandrabani, Dehradun, Uttarakhand, India; 4 Global Tiger Forum, New Delhi, India; Centre for Cellular and Molecular Biology, INDIA

## Abstract

The Sundarban of India and Bangladesh (about 6000 km²) are the only mangrove forests inhabited by a sizeable population of tigers. The adjoining area also supports one of the highest human densities and experiences severe human-tiger conflicts. We used GPS-Satellite and VHF radio-collars on 6 (3 males and 3 female) tigers to study their ranging patterns and habitat preference. The average home range (95% Fixed Kernel) for resident females was 56.4 (SE 5.69) and for males it was 110 (SE 49) km². Tigers crossed an average of 5 water channels > 30 meters per day with a mean width of 54 meters, whereas channels larger than 400 meters were rarely crossed. Tigers spent over 58% of their time within *Phoenix* habitat but compositional analysis showed a habitat preference of the order *Avicennia-Sonneratia* > Phoenix > Ceriops > Barren > Water. Average daily distance moved was 4.6 km (range 0.1–23). Activity of tigers peaked between 05:00 hours and 10:00 hours showing some overlap with human activity. Territory boundaries were demarcated by large channels which tigers intensively patrolled. Extra caution should be taken while fishing or honey collection during early morning in *Avicennia-Sonneratia* and *Phoenix* habitat types along wide channels to reduce human-tiger conflict. Considering home-range core areas as exclusive, tiger density was estimated at 4.6 (SE range 3.6 to 6.7) tigers/100 km^2^ giving a total population of 76 (SE range 59–110) tigers in the Indian Sundarban. Reluctance of tigers to cross wide water channels combined with increasing commercial boat traffic and sea level rise due to climate change pose a real threat of fragmenting the Sundarban tiger population.

## Introduction

The tiger population inhabiting the Sundarban delta forests of India and Bangladesh is one of the largest, isolated tiger populations in the world and unique in its adaptations to a life in the mangrove swamps [1]. Though mangrove habitats are amongst the most productive of ecosystems, they can be an extremely difficult habitat for terrestrial mammals like the tiger and its prey due to lack of fresh water and the difficulties of movement in such swampy terrain. The Sundarban tiger population is well known for two primary reasons: first it is often cited as the largest population of tigers in the world and secondly, for the highest magnitude of human-tiger conflict in the world. According to official records of the Forest Department of India and Bangladesh around 250 and 440 tigers reside in the respective countries [2]. Current estimates based on camera trap data puts this figure to around 180 tigers [3, 4]. In the 1860’s, historical conflict records suggest that 4218 people were killed and eaten by tigers in just six years [5, 6] in Sundarban. About 800 humans were killed by tigers in a span of 20 years between 1950’s and 1970’s in the undivided Sundarban [7]. According to recent estimates, on an average 22 people were killed by tigers annually on the Bangladesh side between 1975 and1999 [8] whereas an average of thirty six humans per year were killed by tigers on the Indian side of Sundarban, with only 28.5% of victims’ bodies being recovered [7].

Baseline information to conserve tigers is lacking for the Indian Sundarban with majority of the studies on tigers and their prey being conducted on the Bangladesh side. Hendrichs [9] correlated human attacks in Bangladesh Sundarban to increasing salinity levels. Other studies to assess prey density were conducted by Reza [10] and Khan [11] in this landscape. Dey [12] carried out a detailed ecological study with special reference to ranging pattern and habitat use by chital and barking deer. Detailed ecological studies on tigers and prey were initiated by a collaborative project between Zoological Society of London and Bangladesh Forest Department in the year 2005, which included monitoring tiger populations in mangrove landscapes [13], designing conservation framework to mitigate human-tiger conflict [14] and studying the impact of sea-level rise on Sundarban [15].

The mangrove habitat of Sundarban provides a formidable challenge for conducting scientific studies, owing to an ever present threat from tigers, absence of roads or trails inside the forests and difficulties in employing standard survey methodologies [16]. Radio telemetry offers insight into certain aspects of tiger ecology that other methodologies, such as secondary sign surveys and camera-trapping, cannot always provide [17]. Tracking individual tigers aids in understanding space use, movement and their likelihood to cross certain habitat features [18, 19]. However, despite its benefits, telemetry studies have only been published from six (8%) of the 76 Tiger Conservation Landscapes (TCL), with majority of the work being from Nepal and Russia [18, 20–29].

Herein, we report home range characteristics, movement pattern and habitat preferences of tigers in Sundarban Tiger Reserve, India, based on Satellite, GPS and VHF telemetry tracking of 6 tigers. The value of the Sundarban for tiger conservation, is due to its large population size and contiguous habitat that is presumably permeable to tigers. In an island landscape mosaic intersected by varying width of water channels, commercial ship traffic and sea level raise are real threats of becoming barriers to free tiger movement across the Sundarban [30]. A thorough investigation of tiger movement will provide insights into potential mitigation measures to prevent tiger population fragmentation. No such data exists for the Sundarban, except for one natural history paper [31]. Herein we use fine resolution GPS telemetry data to evaluate tiger movement across water barriers. Understanding of activity, ranging pattern and habitat use by tigers inhabiting the mangrove swamps could provide information for mitigating human-tiger conflict and formulating conservation oriented management strategies for the entire landscape.

### Methodology

#### Ethics Statement

Permissions for capture and collaring tigers were obtained under the Wildlife (Protection) Act 1972 from the Directorate Wildlife Preservation, Ministry of Environment and Forests, Government of India and the Chief Wildlife Warden, Government of West Bengal. The technical committee of the National Tiger Conservation Authority which also considers the well being of animals and ethics of research approved the research project. Tigers were anesthetized using standard drugs under supervision by qualified veterinarians. All tigers were observed from a safe distance till they fully recovered from the anesthesia and walked away into the forest.

#### Study Site

Sundarban situated at the confluence of the deltas of Brahmaputra, Ganga and Meghna Rivers, is the world’s largest contiguous mangrove forest. The delta spreads across the countries of India and Bangladesh covering 10,000 km^2^ [7] with the Indian part covering about 4,266 km^2^ [32] in the 24 Parganas district of West Bengal. It comprises of mudflats, creeks, tidal channels and an archipelago of about 102 islands of which 54 are inhabited by humans [33] at a density of 1641 per km² [34]. Within this forested tract on the Indian side (1330.12 km²) has been declared as ‘Sundarbans National Park’, designated as the core area of the Sundarban Tiger Reserve, whereas an additional 1255 km² as its buffer zone. The United Nations Educational, Scientific and Cultural Organisation (UNESCO), declared the Indian Sundarban as a World Heritage Site in 1987 for the biological significance of this region, and the unique assemblage of aquatic and terrestrial flora and fauna. Chital (*Axis axis*), wild pig (*Sus scrofa*), rhesus macaque (*Macaca mulatta*), lesser adjutant stork (*Leptoptilos javanicus*) and water monitor (*Varanus salvator*) comprises the principal prey of tiger in the region [35]. Only 1600 km² (64%) of the Sundarban Tiger Reserve is comprised of land while the remaining is comprised of river channels of varying widths. The landscape is characterized by a web of tidal water systems with two high and low tides each day and an average tidal amplitude between 3.5 and 5 meters, when majority (about 70%) of the forests gets inundated [36]. The Sundarban forest is classified under the sub-group 4B tidal swamp forests with subdivisions of mangrove type (4B/TS1 and 4B/TS2), salt water type mixed forest (4B/TS4), brackish type (4B/TS4) and palm swamp type (4B/E1) [37]. Major tree species include *Avicennia alba*, *Avicennia marina*, *Acanthus ilicifolius*, *Aegiceras corniculatum*, *Bruguiera sexangula*, *Ceriops decandra*, *Excoecaria agallocha*, *Nypa fruticans*, *Phoenix paludosa*, *Rhizophora apiculata*, *Sonneratia apetala*, *Xylocarpus granatum* and *Xylocarpus mekongensis* [38].

### Field methods

#### Collaring of tigers

Six tigers (3 adult female and 3 adult males) were radio-collared between 2008 and 2014. The tigers were trapped in cages using bait and tranquilized using 2.5 mg/kg Ketamine and 0.06 mg/kg meditomedine administered intra-muscularly using a blow pipe [39]. The tigers were aged based on eruption and ware pattern of their teeth, development of secondary sexual characters and genital organs. One tigress was equipped with Telonix VHF MOD 400 collar and its 100% home range was reported earlier [40]. The radio-collars (GPS PLUS IRIDIUM, Vectronics Aerospace GMBH, Berlin Germany) weighed less than 1.2% of the body weight of tigers irrespective of the sex. For the 5 tigers, collars were equipped with programmable GPS schedule which recorded fixes between 1–3 hours interval with an IRIDIUM Satellite data uploading facility and a VHF beacon for ground tracking.

#### Vegetation mapping

We used remotely sensed False Color Composite (FCC) maps on 1:50,000 scale from Landsat-5-ETM + cloud free imagery (30M resolution) for the month of February 2010. We ground validated the imagery by sampling 145 circular plots of (15 m radius) along water channels across the tiger reserve by a small single engine boat between February and June 2010. We first used unsupervised maximum likelihood classifier for preparing a vegetation map. Subsequently, supervised classification was done by merging vegetation classes that could be recognized on the ground and made ecological sense. We used 25% of our ground plots to check the accuracy of our vegetation map.

#### Home range of tigers

We used two non-parametric home range estimators: (a) Minimum Convex Polygon (MCP) [41] and (b) Fixed Kernel (FK) [42]. We evaluated the 95% MCP of individual tiger home ranges for comparison with past studies while fixed kernel estimates were used to provide realistic estimates of home ranges and intensively used core areas. The smoothing parameter (h) used for kernel home-range estimates was 1000. Water channels more than a kilometer width were excluded as non habitat from home range size estimates. In theory, home range size estimates reach an asymptote when an adequate sample size of animal locations is reached [43]. Home range size was plotted against the number of locations incremented in groups of 50 locations to assess adequacy of sample size [44, 41]. We estimated fixed kernel home ranges with 5% increment isopleths from 5% to 95% and plotted home range size vs. kernel isopleths. The isopleth at which home range area shows inflection was identified as the isopleth depicting the core area of the home range [45]. Radio-telemetry data from over twenty tigers in other parts of India suggest that the core area of home ranges are exclusive with minimal overlap between individuals while outer parts of tiger home ranges could overlap with neighbors substantially (YVJ & QQ unpub. data). Based on this observation average core area was considered to be exclusive area of home range, and we used this to obtain a crude estimate of the density and population size of tigers for the entire Sundarban Tiger Reserve.

#### Effect of tide and day on activity of tigers

We selected only those 24 hr periods (days) that had a minimum of 12 GPS fixes to derive estimates of daily travel distance (n = 222 full 24 hr periods) from 5 collared tigers. For each day, the distances between sequential GPS fixes were measured and added up to calculate total distance travelled. Distance moved between two successive locations were estimated by using Hawth’s Tool in Arc GIS 9.3 [46]. We depict daily activity pattern of tigers based on hourly distance moved using a histogram. Sundarban is greatly affected by oceanic tides. At peak high tide over 70% of the area is covered by water and all mudflats are water logged. Tidal currents and water extent would likely govern tiger movement in these habitats. Spring tide during which tidal fluctuations and currents are extreme, lasts for eight days followed by Neap tide phase for six days during which time the tide effects are milder. We consulted Lunar Calendars to classify daily distance moved by tigers during Neap and Spring tidal phases. Day and night time were defined as even 12 hour periods from 6AM to 6PM and 6PM to 6AM respectively. Movement of tigers could potentially be different between day and night time as well as differ between neap tide time and spring tide duration. After normalizing tiger distance data by a log transformation, we analyzed the log of distance moved by individual tigers using a linear mixed effect model where time was considered as night and day while effect of tide was considered as spring tide and neap tide. Variation due to individual tigers was considered as a random error in the model. The analysis was done in R 3.2.2 software using the function lme in the package "lme4" [47].

#### Channels crossed by tiger

We digitized the daily trajectories of individual tigers overlaid on Google Earth Pro imagery to determine the number and width of channels crossed. Considering all radio locations from all tigers, a common minimum convex polygon was created using Arc GIS 9.3 [46]. We generated 5 random trajectories for each tiger trajectory within the common polygon using Hawth’s Tool in Arc GIS 9.3 (Animal Movement CRW Models) [46] (S1 Fig). The average number of steps in all original tiger trajectories was used to generate the corresponding random trajectories. At each step node the random trajectory could extend further in any direction. A minimum and maximum step length was provided for each trajectory based on the original trajectory. Trajectories which extended outside the study area polygon or ended in water were discarded and a new random trajectory generated in its place. Water channels were classified by 50 meter width intervals up to 1000 meters, and then into 500 meter width intervals. Availability of channels of varying width for tigers to cross in the landscape was determined by the number of channels in each width category traversed by random trajectories. These were compared subsequently with the actual number of channels crossed by tigers from the 24 hr movement trajectories by chi-square test [48] in NCSS 10.0 [49] to test the hypothesis that tigers crossed water channels in proportion to their availability and that large water channels were not barriers to their movement.

#### Resource selection and habitat use

Habitat use by tigers was estimated as the percent number of locations in each habitat type [50, 51]. We considered 95% FK home range as a representation of the total area from within which an animal had the opportunity to choose different habitat types. Therefore, availability of different habitat types to a tiger was computed as the area of a habitat within its 95% FK home range in a GIS domain [52]. Habitat preference of tigers was computed using compositional analysis [50]. Each tiger was considered as a sample for statistical analysis [53]. Habitat preference of each individual tiger within its 95% FK home-range was also calculated using Ivlev’s Index [54] for graphical presentation.

## Results

### Habitat map

**Fig 1:**
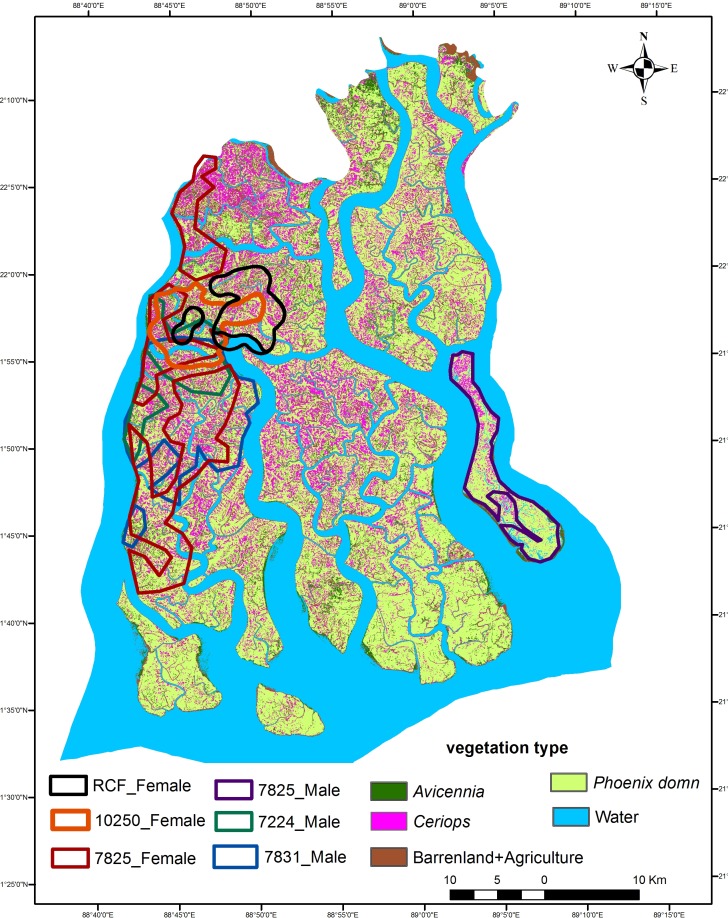
Home ranges (95% Fixed Kernel) of radio-collared tigers superimposed on the classified habitat map of Sundarban Tiger Reserve, India.

We identified five habitat classes based on ground cover, (Fig 1) these were:

1. Water/Channels- This category consisted of open water and inland water channels exceeding 20m in width. This habitat comprised of 1161.44 km² (41.48%) of the study area.
2. *Phoenix dominated*- The habitat was predominantly of *Phoenix paludosa* along with *Excoecaria agallocha* forming dense thickets on high lands with compact soil and minimum inundation during high tides. This habitat therefore represents shady but thorny palms on drier ground and covered 1033.76 km² (36.92%) of the study area.
3. *Ceriops dominated*- *Ceriops decandra* was the dominant mangrove species here. It formed dense shrub vegetation of moderate height growing on comparatively higher ground. The habitat covered 333.48 km² (11.91%) of the study site.
4. Barren dry areas covered 145.6 km² (5.2%) They were open areas that occasionally get tidal influx. This results in hard salt encrustation of the ground and low water availability. Vegetation cover was sparse.
5. *Avicennia-Sonneratia* habitats covered 126.56 km² (4.52%) and were primarily low lying areas with fresh deposition of silt that are characterized by *Avicennia* species and flanked by the foreshore grasslands of *Oryza coarctata*. *Sonneratia apetala* also grows along with *Avicennia* along banks of these fertile, moist and mostly flat lands.

Based on classification of the validation plots, the accuracy of the habitat map was determined to be 80%.

### Home ranges of tigers

The radio-collar on the tiger 7224M dropped off after 18 days. Therefore Data from 7224M and the transient tigress 7825 were not used for computing average home-range. The number of tracking days for four individual tigers varied from 59 to 206. The fix acquisition success rate was 66.6% (SE 5.85) with an average of 12 fixes obtained per day.

On the average home ranges reached an asymptote at 430 fixes (S2 Fig). The estimated average tiger home range (95% FK) size was 162 km² (SE 37) (range 61-191km²) (Table 1). The average male home range (n = 2) (95% FK) was 110km² (SE 49) (range 79–154 km²). Tigress 7825F being a transient had a large home range (Fig 1). Tigress 10250 and RC Female had (95% FK) home ranges of 62km² and 51km² respectively. The average female home range (n = 2) (95% FK) was 57 (SE 6) km² (Table 1, Fig 1). The isopleth of fixed kernel that defined the core area of home ranges was estimated at 75% (S3 Fig). The average home range core area of tigers was estimated to be 60 (SE 13) km². The average core area home range of male (n = 2) was 58 (SE 25.51) whereas for females (n = 2) it was 35 (SE 7.25) km² respectively. Taking into consideration the overall land area of the tiger reserve i.e. 1645 km² and the average core area home ranges, the total number of females and males that could be accommodated would be about 47 (standard error range 39–60) and 28 (standard error range 20–50) respectively, this would give a territorial tiger density of about 4.6 (standard error range 3.6–6.7) tigers per 100 km² for the entire tiger reserve.

**Table 1:** Home ranges of radio-collared tigers as determined by minimum convex polygon (MCP) and fixed kernel (FK) estimators in the Mangrove forests of the Sundarban Tiger Reserve, India.

Tigers	95% MCP km²	95% FK km²	75% FK km²	Total Number of Tracking Days	Total Fixes
**7825 Male**	79.29	60.97	32.76	76	928
**7831 Male**	153.85	159.09	83.78	176	2532
**Mean Male HR**	116.57	110.03	58.27	126	1730
*7224 Male	78.62	68.7	33.44	18	122
**Standard Error**	37.28	49.06	25.51	50	802
**10250 Female**	67.64	62.17	42.06	206	1335
**RCFemale**	35.02	50.80	27.55	14	38
**Mean Female HR**	51.33	56.49	34.81	110	686.5
**Standard Error**	16.31	5.69	7.26	96	648.5
#**7825 Female**	309.67	191.31	79.46	59	643
**Mean Overall**	168.59	162.36	59.52	129	927.67
**Standard Error**	56.66	36.99	12.93	36.34	372.58

*Not used for computing the mean HR as number of tracking days were too few.

#Not used for computing mean female HR as she was a dispersing individual.

### Daily Travel Distances

**Fig 2:**
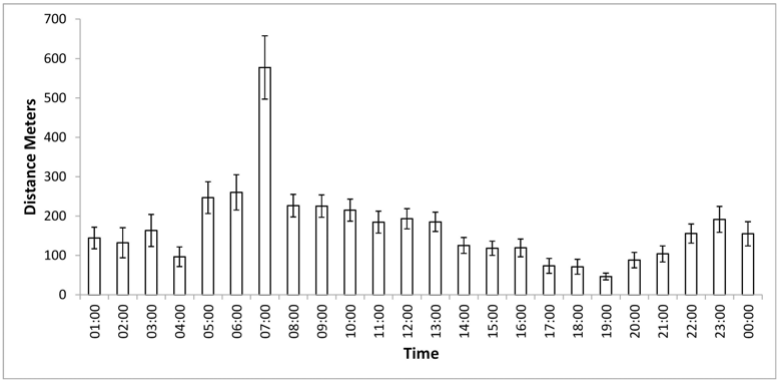
Average distance moved per hour by radio-collared tigers during different time zones in Sundarban Tiger Reserve.

The average distance moved by tigers per day was 4.6 (SE 1.11) range (0.1–23) km. Tiger 7224 M moved 8.12 (SE 3.27) km per day while tigress 10250 moved 1.25 (SE 0.15) km per day. Male tigers moved an average distance of 4.65 km (SE 0.33), whereas females moved 2.91 km (SE 0.39) per day. The transient female 7825 F moved an average distance of 4.86 km (SE 0.66) per day. After accounting for the effect of individual tigers as a random error, we found no effect of day time (t = -1.712, p = 0.09) or tide period (t = -1.165, p = 0.25) on tiger movement (S4 Fig). Tigers were observed to be most active between 5 to 10 AM with a pronounced peak around 7 AM (Fig 2). Radio-collared tigers were observed to achieve speeds of over 5 km per hour on six occasions during 222 days tracking data.

### Channels crossed by tigers

**Fig 3:**
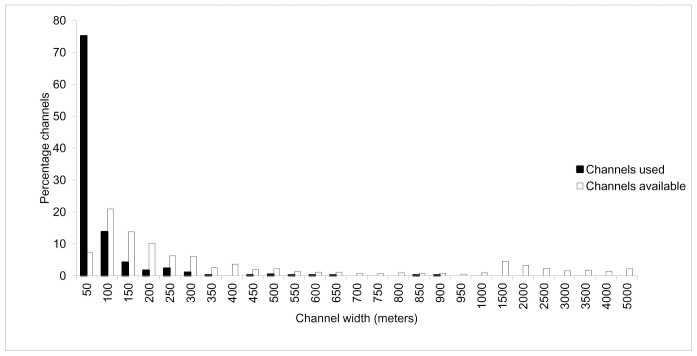
Availability of different width channels in Sundarban Tiger Reserve and the percent frequency of tigers crossing these channels.

Tigers did not cross different width water channels as per the frequency of encountering them (χ² = 676.94, df = 27, P < 0.00001) (S5 Fig), larger channels > 400 meter in width were seldom crossed (Fig 3). Female 7825 the transient tigress and Male 7825 occasionally crossed channels over 400 meter in width, whereas rest of the tigers were not recorded to cross > 400 meter channels. The mean number of channels crossed by tigers per day was 4.8 (SE 1.35).

### Habitat use and preference

Compositional analysis showed that tigers exhibited habitat preference (χ^2^ = 15.01, df = 4, p < 0.05). The order of preference was as follows:

*Avicennia-Sonneratia* habitat > *Phoenix* dominated habitat > *Ceriops* dominated habitat > Barren areas > Water.

**Fig 4:**
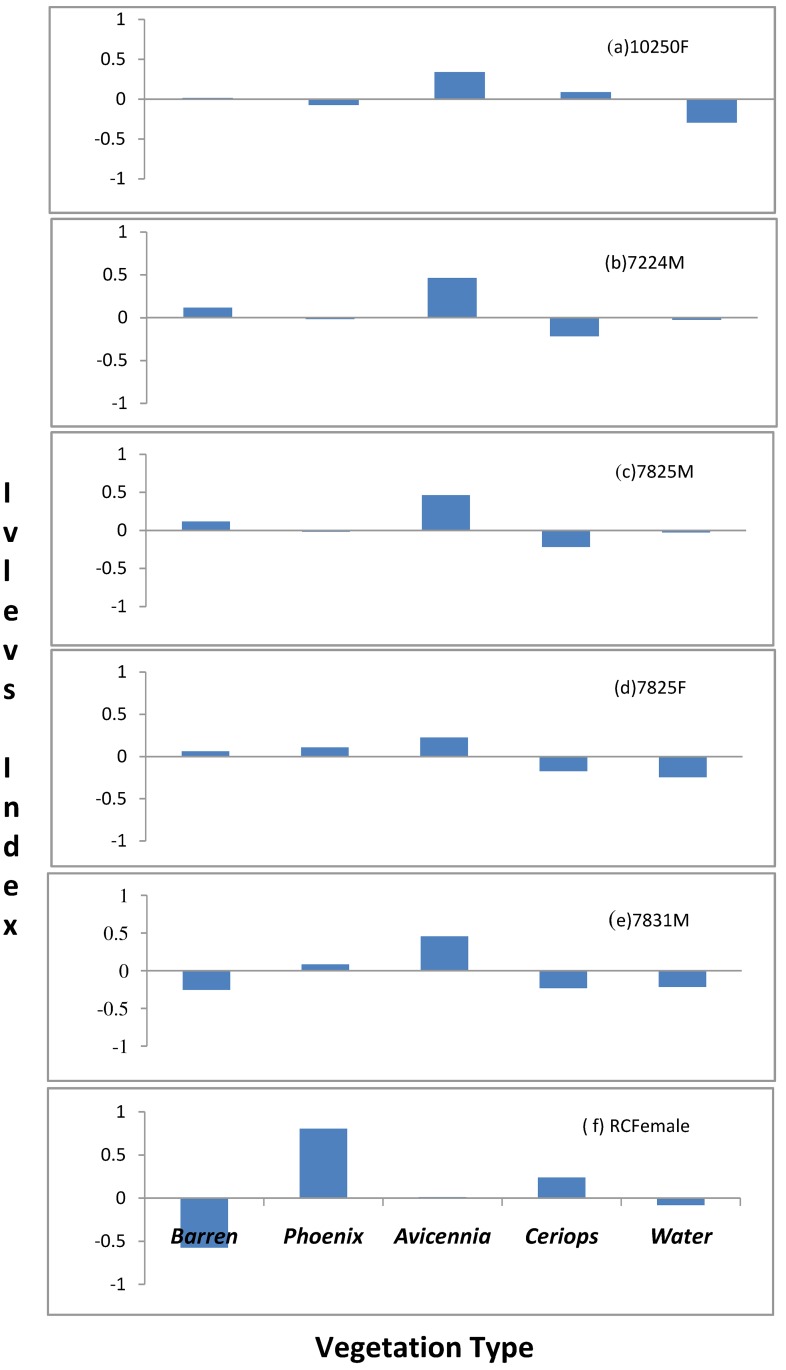
Habitat preference of individual tigers (n = 6) as depicted by Ivlev’s Index.

Though *Avicennia-Sonneratia* was preferred, 58% of tiger locations were in the most common habitat type (*Phoenix dominated areas*) followed by 18% within *Ceriops* dominated habitats. As expected tigers avoided water. Ivlev’s index for individual tigers too showed a preference for *Avicennia* and *Phoenix* followed by *Ceriops* habitat types, in most cases similar to the results of the compositional analysis (Fig 4).

## Discussion

Radio collared tigers were often observed to achieve speeds of over 5 km per hour during movement peaks. Therefore, tigers were capable of being in any part of their home range within an hour. Our one to three hourly sampling scheme for the GPS schedule on the collars provided fine resolution movement data as well as minimized effects of serial autocorrelation in subsequent locations.

Male Tigers compete for access to resources, primarily mates and maintain exclusive home ranges encompassing home ranges of one to several females [21]. Whereas, female tigers maintain home ranges sufficiently large to meet the energetic demands of rearing cubs [22, 55]. Size of home ranges show an inverse relationship with abundance of prey [56, 57]. The smallest ranges reported are from tropical deciduous forests and resource rich grasslands of India that have high primary productivity and support high ungulate abundance and diversity. Here, home range as small as 25 km² for males and 10 km² for females have been reported from Nagarahole [58], Kanha [29] and alluvial floodplains of Chitwan, Nepal [21, 22, 26]. Telemetry studies have shown that breeding tigers are territorial with an average home range size of 12.3 km² in Bangladesh Sundarban [59], 40 km² in Indian Sundarban [40]. The largest home range of tigers, 1385 km² for males and 390 km² for females are reported from the Russian Far East, a result of low prey abundance [60]. Range size of Indian Sundarban tigers were between these extremes and suggests higher resource availability compared to the Russian Far East but lower than that of deciduous forests and alluvial grasslands [61]. This study shows that range sizes are much larger than reported from Bangladesh Sundarban [62] suggestive of a low tiger density on the Indian side. The density estimates of 4.6 tigers per 100 km² derived from this radio-telemetry study is similar to the camera trap based estimates of 4.3 (SE 0.3) tigers per 100 km² reported earlier [63]. However, these should be considered only as crude estimates as they do not account for the dispersal age segment of the population and also considers core areas of territorial tigers to be adjoining, which may not be the case in nature.

Due to low sample of collared tigers, individual tigers had profound effect on mean values. We therefore did not use the range estimates from the single transient female in computing mean home size. This transient tigress was captured on the edge of the mangrove forest near a village. She had a home range of about 310 km² (95% MCP) which is comparable to the home range of a dispersing tigress (726 km², 95% MCP) in a human dominated landscape near Nagpur, Maharashtra [64]. This tigress travelled a minimum distance of 120 kilometers in 27 days. Male 7825 captured from near one of the peripheral villages on the northern part of the Tiger Reserve crossed an 800 meter wide Harinbhanga River in early June 2010 and settled in Talpatti island of Bangladesh, suggesting that though tigers prefer not to cross wide water channels these were not barriers to their dispersal.

The tiger population of India and Bangladesh Sundarban is a single continuous population and trans-boundary conservation between the two countries is vital for long-term population persistence. With increasing commercial boat traffic across wide water channels especially along the International border and in some water channels of Bangladesh, these wide channels could become barriers to tiger gene flow, effectively fragmenting the population of Sundarban tigers into smaller isolated populations. Careful planning and management of “tiger corridors” across Sundarban is required to maintain the Sundarban tiger population as a contiguous population with gene flow between larger islands and between the two countries.

The mean distance moved by tigers per day in the present study was comparatively more than that reported earlier from Bangladesh (3.6 km/day) [62], Panna 1.4 km/day [24] and Chitwan (2.4 km/day) [21] but lower than that reported from Russia (6.4 km/day) [55]. This could be an artifact of sampling since earlier studies relied on VHF telemetry while the current study used GPS-Satellite collars with a high sampling intensity (hourly location schedule) providing more realistic movement data. VHF transmitters though being relatively inexpensive, tracking of animals is comparatively time and labour-intensive [65] and the precision of locations is poor as it is obtained using triangulation [66]. GPS collars in contrast provide the additional benefits of changeable programming of a location schedule, high frequency of locations to derive movement rates along with temporal and spatial precision of positioning data [67]. Usage of GPS collars thus reveal details such as step lengths and turning angles of individual animal trajectories, choice of specific habitat features especially of secretive large carnivores occupying inaccessible areas which can be used to assist conservation programs [68]. Besides the effect of better telemetry technology on estimates of distance measures, the large distance observed to be moved by tigers could also be in response to low prey abundance in the Sundarban [69] as also seen in the Russian Far East.

It appears that tigers moved most during dawn and early morning hours. Sunrise in the Sundarban region was between 4:45 to 6 AM and tiger activity coincided with this period. Tigers are crepuscular and nocturnal elsewhere, an adaptation to evade confrontation with humans in majority of landscapes where they co-exist [70] and in synchrony with the activity of their principal prey sambar [71, 72]. Livestock rearing, which brings tigers in conflict with humans resulting in their persecution was historically not a livelihood option within the Sundarban mangroves. Also *shikar* (hunts) of tigers was rarely an organized activity in the Sundarban compared to other parts of India due to the hostile nature of the habitat and prevalence of disease [73]. Due to this Sundarban Tigers are likely to be least persecuted and therefore may not relate to people with fear and possibly even consider them as potential prey. Also the primary prey of the tiger, the spotted deer is diurnal with activity peaks during morning and evening [71, 74]. These two factors possibly explain the higher activity of the Sundarban tiger during morning hours. The majority of human kills by tigers were recorded in the daytime between 7 AM and 12 PM [7, 75] suggesting that if human activity were reduced in the early morning hours, lethal attacks by tigers could potentially be reduced.

Tigers routinely commute between islands either in search of prey or patrolling territories with an average of 5.19 channels crossed per day and a mean width of 54 meters (SE 4.1). Though channels of more than 400 meters in width were seldom crossed, banks of these wide channels were intensively patrolled by tigers and likely form natural boundaries for home ranges (S6 Fig). Since shores of wide channels are used frequently by tigers, encounters with humans (fishermen, honey collectors and crab collectors) and small boats anchored along the shore of such channels would be more compared to smaller channels or inland. *Avicennia and Sonneratia* patches were found along channel banks in relatively low lands, with regular tidal water inundation. These habitats were preferred by chital and macaques due to their higher food value [12]. The preference for these habitats by tigers is possibly explained by the higher availability of prey and ease of hunting in these habitats.

Tigers also spend considerable time within *Phoenix* and *Ceriops* dominated patches which offer shelter from tidal inundations since these habitats are on relatively higher ground and might therefore be often used as resting and cub rearing refuges. Tigers were reported to have a preference for *Phoenix* and *Sonneratia*-*Avicennia* mixed stands in the Indian Sundarban [75, 76], similar to the present findings. Honey and crab collectors who venture inside *Avicennia-Sonneratia* and *Phoenix* dominated patches should take utmost caution to prevent fatal encounters.

The Sundarban Tiger Population is of global importance for the conservation of the species due to the population size, contiguous nature of the habitat and the unique adaptations of the tigers that inhabit these mangrove forests. In spite of the small number of collared tigers our study contributes by enhancing our understanding of tiger ecology in this unique habitat. We now know that range sizes are likely larger than reported earlier from Bangladesh [62]. Assuming 95% FK female home ranges as exclusive area and a demographic ratio of tigers from Chitwan Nepal, Barlow [62] estimated the density of tigers to be 23.5 tigers per 100 km² giving a population estimate of around 335 to 1000 tigers for the entire landmass of Bangladesh Sundarban. However, data from the two radio-collared tigress used in that study was of short duration and the tigress occupied areas of high prey density in the south-eastern part of Bangladesh Sundarban. Thus, population estimates derived based on this data seems likely to be an overestimate and the present study provides more realistic information on home ranges suggestive of a lower tiger density of 4.6 tigers per 100 km² for the Indian side.

Our findings suggest that Sundarban tigers are primarily active during morning, prefer certain habitat types and are reluctant to cross wide expanse of water. These have far reaching implications that can be used to minimize human-tiger conflict which is a major impediment to tiger conservation in this region. In light of rising sea levels due to climate change [15] combined with increased commercial ship traffic within water channels of the Sundarban, there is a real threat that this hereto large tiger habitat will become fragmented, restricting gene flow between islands. International cooperation between India and Bangladesh is required for identification and management of optimal tiger movement corridors, wherein ship traffic would need to be regulated. Mitigating sea level rise would be more difficult, but needs to be addressed as a major threat to the future persistence of these uniquely adapted tigers [15]. It seems likely that with rising sea levels, mangrove forests will be submerged and destroyed or shift inland, ingressing into fertile agricultural fields that currently support high human density. The Governments of India and Bangladesh need to be ready to address the problem of human resettlement, develop alternative livelihood options and deal with enhanced tiger-human conflict. The Sundarban is a dynamic system with siltation and tidal erosion working in unison to create new islands and submerging old islands continuously [15]. Since natural processes are inherently slow, management interventions that enhance the rate of colonization by mangroves to stabilize islands and increase rates of silt deposition would help combat sea level rise to some extent [15]. Tigers are adaptable species, but in this case they need human intervention to combat manmade alterations to their ecosystem.

## Supporting Information

S1 Fig Daily Tiger Paths (n = 222) and simulated random paths (n = 674) from 5 radio-collared tigers with Global Positioning based fixes every 1 hr overlaid on the Google Earth Image of the study area of Sundarban Tiger Reserve. (TIFF)

S2 Fig Plot of Minimum Convex Polygon home ranges of radio-collared tigers against number of fixes in Sundarban Tiger Reserve to determine adequacy of fixes (sample size) for home range estimation. (TIFF)

S3 Fig Plot of home range size (km²) as a function of declining fixed kernel isopleths of five tigers. The point of inflection of the curve depicts the isopleth that best describes the core area of the home range, Error bars are standard errors.
(TIFF)

S4 Fig Distance moved by five radio-collared tigers during day and night time and effect of tide phase shown by box & whisker plot. (TIFF)

S5 Fig Availability and Crossing of different width of channels (meters) by radio-collared tigers shown by box & whisker plot. The data has been presented on a log scale. The bottom of the box indicates the 25th percentile. The top of the box represents the 75th percentile. The points outside the box are outliers. The asterisks or stars are extreme outliers. These represent cases/rows that have values more than three times the height of the boxes.
(TIFF)

S6 Fig Movement paths of tiger 7825M. The path highlights the patrolling of shores of wide water channels by the tiger on Talpatti Island of Bangladesh.
(TIFF)

## References

[1] Dinerstein E, Wikramanayake ED, Robinson JG, Karanth KU, Rabinowitz A, Olson D, et al A framework for identifying high priority areas and actions for the conservation of tigers in the wild World Wildlife Fund-US and Wildlife Conservation Society. Published in Association with the National Fish and Wildlife Foundation’s Save the Tiger Fund; 1997.

[2] Global Tiger Initiative Secretariat. Global Tiger Recovery Program Implementation Report. The World Bank, Washington, DC; 2012.

[3] Jhala YV, Qureshi Q, Gopal R. The status of tigers in India In: Jhala YV, Qureshi Q, Gopal R editors. National Tiger Conservation Authority, New Delhi and the Wildlife Institute of India, Dehradun: 2014.

[4] Dey T, Kabir MdJ, Ahsan MdM, Islam M, Chowdhury MdMR, Hassan MdS, et al. Tiger Abundance in Bangladesh Sundarbans. Technical report by the Bangladesh Forest Department Dhaka and the Wildlife Institute of India, Dehradun; 2015.

[5] Montgomery S. Spell of the tiger: the man-eaters of Sundarbans Vermont Chelsea Green Publishing; 2008.

[6] Blanford WT. Fauna of British India, Mammalia. Taylor and Francis, London; 1891.

[7] Chakrabarti K. Man-eating tigers Darbari Prokashan, Calcutta; 1992.

[8] Reza AHMA. Ecology of the Bengal tiger *(Panthera tigris tigris)* in the Sundarban. M.Sc. Thesis, Jahangirnagar University, Savar, Dhaka, Bangladesh. 2000.

[9] Hendrichs H. The status of the tiger (*Panthera tigris)* in the Sundarban mangrove forest (Bay of Bengal). Saugetierk Mitt. 1975; 23: 161–199.

[10] Reza AHMA, Feroz MM, Islam MA. Man-tiger interaction in the Bangladesh Sundarban. Bangladesh J of Lf Sci (1 & 2), 2002; 14: 75–82.

[11] Khan MMH. Ecology and conservation of the Bengal tiger in the Sundarbans Mangrove forest of Bangladesh. PhD thesis, University of Cambridge. 2004.

[12] Dey TK. Population ecology of the barking deer (*Muntiacus muntjak*) and spotted deer (*Axis axis*) of Bangladesh. PhD Thesis, Department of Zoology, University of Dhaka. 2004.

[13] Barlow ACD, Ahmed MIU, Rahman MM, Howlader A, Smith AC, Smith JLD. Linking monitoring and intervention for improved management of tigers in the Sundarbans of Bangladesh. Biol Conserv. 2008; 141: 2032–2040.

[14] Barlow ACD, Greenwood CJ, Ahmad IU, Smith JLD. Use of an Action-Selection Framework for Human-Carnivore Conflict in the Bangladesh Sundarbans. Conserv Biol. 2010; 24: 1338–1347. doi: 10.1111/j.1523-1739.2010.01496.x 20345402 10.1111/j.1523-1739.2010.01496.x

[15] Loucks C, Meyer BS, Hossain AAMd, Barlow ACD, Chowdhury MR. Sea level rise and tigers: predicted impacts to Bangladesh’s Sundarban mangroves. Clim Change. 2010; 98: 291–298.

[16] Karanth KU, Nichols JD. Estimating tiger densities in India from camera trap data using photographic captures and recaptures. Ecol. 1998; 79: 2852–2862.

[17] Goodrich JM, Miquelle DG. Translocation of problem Amur tigers (*Panthera tigris altaica)* to alleviate tiger-human conflicts. Oryx. 2005; 39: 454–457.

[18] Smith JLD. The role of dispersal in structuring the Chitwan tiger population. Behv. 1993; 124: 165–195.

[19] Smith JLD, Ahearn SC, McDougal C. Landscape analysis of tiger distribution and habitat quality in Nepal. Conserv Biol. 1998; 12: 1338–1346.

[20] Seidensticker J. On the ecological separation between tigers and leopards. Biotr. 1976; 8: 225–234.

[21] Sunquist ME. The social organization of tigers (*Panthera tigris*) in Royal Chitwan National Park, Nepal Washington, DC: Smithsonian Institution Press; 1981.

[22] Smith JLD, Mcdougal CW, Sunquist ME. Land tenure system in female tigers Tigers of the world: the biology, biopolitics, management, and conservation of an endangered species. In: Tilson RL, Seal US editors. New Jersey: Noyes Publications, Park Ridge; 1987 pp. 97–108.

[23] Kotwal PC, Gopal R. Radio-telemetry and field observations on territoriality of tigers in Kanha National Park. Tiger Paper. 1995; 22: 6–11.

[24] Chundawat RS, Gogate N, Johnsingh AJT. Tigers in Panna: preliminary results from an Indian tropical dry forest In: Seidensticker J, Christie S, Jackson P editors. Riding the tiger: tiger conservation in human-dominated landscapes Cambridge, Cambridge University Press; 1999 pp. 123–129.

[25] Miquelle DG, Quigley HB, Smirnov EN, Merrill T, Myslenko AE, Hornocker MG, et al Hierarchical Spatial Analysis of Amur Tiger Relationships to Habitat and Prey In: Christie S, Seidensticker J, Jackson P editors. Riding the Tiger: Tiger Conservation in Human-dominated Landscape, Cambridge University Press; 1999 pp. 71–99.

[26] Karanth KU, Sunquist ME. Behavioural correlates of predation by tiger (*Panthera tigris*), leopard (*Panthera pardus*) and dhole (*Cuon alpinus*) in Nagarahole, India. Zool. 2000; 250: 255–265.

[27] Kerley LL, Goodrich JM, Miquelle DG, Smirnov EN, Nikolaev IG, Quigley HB, et al Reproductive parameters of wild female Amur (Siberian) tigers (*Panthera tigris altaica*). Mamm. 2003; 84: 288–298.

[28] Goodrich JM, Miquelle DG, Smirnov EN, Kerley LL, Quigley HB, Hornocker MG, et al Survival rates and causes of mortality of Amur tigers on and near the Sikhote-Alin Biosphere Zapovednik. Zool. 2008; 276: 323–329.

[29] Sharma RK, Jhala YV, Qureshi Q, Vattakaven J, Gopal R, Nayak K. Evaluating capture-recapture population and density estimation of tigers in a population with known parameters. Anim Conserv. 2010; 1: 94–103.

[30] Smith JLD, McDougal CW, Miquelle DG. Chemical communication in free ranging tigers (*Panthera tigris*). Animal Behaviour 1998; 37: 1–10.

[31] Garga DP. How far can a tiger swim? Journal of the Bombay Natural History Society 1947; 47: 545–546.

[32] Sen N, Naskar K. Algal flora of Sundarbans Mangal Daya Publishing House, Delhi, 2003.

[33] Bera GK, Sahay VS. In the lagoons of the Gangetic delta Mittal Publications, New Delhi, 2010.

[34] Anon 2011. District Population Census 2011, West Bengal literacy sex ratio and density Census of India Available: http://www.census2011.co.in/census/district/11-north-twenty-four-parganas.html; http://www.census2011.co.in/census/district/17-south-twenty-four-parganas.html

[35] Khan MMH. Prey selection by tigers (*Panthera tigris)* in the Sundarbans West Wildlife Sanctuary of Bangladesh. BNHS. 2008; 3: 105.

[36] Hazra S, Ghosh T, Das Gupta R, Sen G. Sea level and associated changes in the Sundarbans. Science and Culture 2002; 68: pp. 309–321.

[37] Champion H, Seth S. A revised study of the forest types of India New Delhi: Government of India Press; 1968: pp. 404.

[38] Gopal B, Chauhan M. Biodiversity and its conservation in the Sundarban mangrove ecosystem. Aquatic Sciences–Research across boundaries, 2006; 68 (3): 338–354.

[39] Kreeger TK. Handbook of Wildlife Chemical immobilization International Wildlife Vet. Services Inc. Post Box 37, Larammie, WY, USA, 1996.

[40] Sharma RK, Jhala YV, Qureshi Q. Home range size of a tigress in Sundarbans, India: preliminary results. CATNEWS. 2011; 54: 13–16.

[41] Mohr CO, Stumpf WA. Comparison of methods for calculating areas of animal activity. Wildl Manag. 1996; 30: 293–304.

[42] Worton BJ. Kernel methods for estimating the utility distribution in home-range studies. Ecol. 1989; 70: 164–168.

[43] Harris S, Cresswell WJ, Forde PG, Trewhella WJ, Wollard T, Wray S. Home–range analysis using radio–tracking data: a review of problems and techniques applied to the study of mammals. Mammal Rev. 1990; 20: 97–123.

[44] Kernohan BJ, Gitzen RA, Millspaugh JJ. Analysis of animal space use and movements In: Millspaugh JJ, Marzluff JM editors. Radio tracking and animal populations. London: Academic Press; 2001 pp. 125–166.

[45] Powell RA. Animal home ranges and territories and home range estimators In: Boitani L, Fuller TK editors. Research techniques in animal ecology: controversies and consequences. Columbia University, New York, 2000 pp. 65–110.

[46] Rodgers AR, Kie JG. HRT: Home Range Tools for Arc GIS, version 1.1, 24.4.2011, 2010.

[47] Bates D, Maechler M, Bolker B, Walker S, Cristensen RHB, Singmann H, et al. Package ‘Lme4’. Available: http://cran.r-project.org/web/packages/lme4/index.html. 2012

[48] Zar HJ. Biostatistical Analysis, Fourth Edition, Pearson Prentice Hall, Dorling Kindersley Publishing Inc, Upper Saddle River, NJ; 2009.

[49] NCSS 1997®; NCSS Statistical software, Kaysville, Utah.

[50] Aebischer NJ, Robertson PA, Kenward RE. Compositional analysis of habitat use from animal radio tracking data. Ecol. 1993; 74: 1313–1325.

[51] White GC, Garrot RA. Analysis of wildlife radio-tracking data USA: Academic Press; 1990 pp 383.

[52] Hooge PN, Eichenlaub B. Animal movement extension to Arcview. Version 2.0. 2000 Alaska science centre—biological science office. US Geological Survey, Anchorage, USA.

[53] Garton EO, Wisdom MJ, Leban FA, Johnson BK. Experimental design for radio-telemetry studies In: Millspaugh JJ, Marzluff JM editors. Radio tracking and animal populations. London: Academic Press; 2001 pp. 15–42.

[54] Krebs CJ. Ecological Methodology. Harper Collins Publishers, New York; 1989.

[55] Miller CS, Hebblewhite M, Petrunenko YK, Seryodkin IV, Goodrich JM, Miquelle DG. Amur tiger (*Panthera tigris altaica*) energetic requirements: Implications for conserving wild tigers, Biol Conserv 2014; 170: 120–129.

[56] Miquelle DG, Goodrich JM, Smirnov EN, Stephens PA, Zaumyslova O, Yu-Chapron G, et al The Amur Tiger: a case study of living on the edge In: MacDonald DW, Loveridge A. editors. Biology and Conservation of Wild Felids. Oxford UK, Oxford University Press; 2010 pp. 325–339.

[57] Simcharoen A, Savini T, Gale GA, Simcharoen S, Duangchantrasiri S, Pakpien S et al Female tiger (*Panthera tigris*) home range size and prey abundance: important metrics for management. Oryx. 2014; 48: 370–377.

[58] Karanth KU, Sunquist ME. Prey selection by tiger, leopard and dhole in tropical forests. Anim Ecol. 1995; 64: 439–450.

[59] Barlow ACD, Smith JLD, Ahmad IU, Hossain ANM, Rahman M, Howlader A. Female tiger (*Panthera tigris*) home range size in the Bangladesh Sundarbans: the value of this mangrove ecosystem for the species’ conservation. Fauna and Flora International. Oryx. 2011; 45 125–128.

[60] Goodrich JM, Miquelle DG, Smirnov EN, Kerley LL, Quigley HB, Hornocker MG. Spatial structure of Amur (Siberian) tigers (*Panthera tigris altaica*) on Sikhote–Alin Biosphere Zapovednik, Russia. Mammal. 2010; 91: 737–748.

[61] Karanth KU, Nichols JD, Kumar NS, Link WA, Hines JE. Tigers and their prey: predicting carnivore densities from prey abundance. Proceedings of Natural Academy of Science. 2004; 101: 4854–4858. 10.1073/pnas.0306210101 PMC387338 15041746

[62] Barlow ACD. The Sundarbans tiger: Adaptation, population status and conflict management. PhD thesis, University of Minnesota, Saint Paul, USA. 2009.

[63] Jhala YV, Qureshi Q, Gopal R, Sinha PR. Status of the Tigers, Co-predators and Prey in India. In: Jhala YV, Qureshi Q, Gopal R and Sinha PR editors. National Tiger Conservation Authority, Govt. of India, New Delhi and Wildlife Institute of India, Dehradun, 2011.

[64] Athreya V, Navya R, Punjabi GA, Linnell JDC, Odden M, Khetarpal S, et al Movement and activity pattern of a collared tigress in a human-dominated landscape in central India. Trop Conserv Scn. 2014; 7: 75–86.

[65] Rodgers AR. In: Millspaugh JJ, Marzluff JM editors. Radio tracking and animal populations London: Academic Press; 2001 pp. 79–121.

[66] Withey JC, Bloxton TD, Marzluff JM. Effects of tagging and location error in wildlife radiotelemetry studies In: Millspaugh JJ, Marzluff JM editors. Radio tracking and animal populations. London: Academic Press; 2001 pp. 45–69.

[67] Meade J, Biro D, Guilford T. Homing pigeons develop local route stereotypy. Proceedings of the Royal Society B-Biological Sciences 2005; 272: 17–23. 10.1098/rspb.2004.2873 PMC1634935 15875565

[68] Calenge C, Dray S, Royer-Carenzi M. The concept of animals' trajectories from a data analysis perspective. Ecol. Informatics 2009; 4: 34–41.

[69] Roy M, Qureshi Q, Naha D, Sankar K, Gopal R, Jhala YV. Demystifying the Sundarban tiger: novel applications of conventional population estimation methods in a unique ecosystem. Popul Ecol. 2015 (In Press)

[70] Inskip C, Zimmermann A. Human–felid conflict: a review of patterns and priorities worldwide. Oryx. 2009; 43: 18–34.

[71] Schaller GB. The deer and the tiger University of Chicago Press, Chicago; 1967.

[72] Kawanishi K, Sunquist ME. Conservation status of tigers in a primary rainforest of Peninsular Malaysia. Biol Conserv. 2004; 120: 329–344.

[73] Simson BF. Rhinos, Javan and Indian In: Rangarajan M editor. The Oxford Anthology of Indian Wildlife Volume I, Hunting and Shooting. New Delhi: Oxford University Press; 1999 pp 58–59.

[74] Tak PC, Lamba BS. Ecology and ethology of the spotted deer, *Axis axis axis* (Erxleben) (Artiodactyla: Cervidae). Records of the Zoological Survey of India, Occasional Paper 1984. No. 43: 100.

[75] Chakrabarti K. Statistical ecology of Sundarbans tiger. Tiger Paper. 1984; 11: 29–31.

[76] Chowdhury MK, Sanyal P. Man-eating behaviour of the tigers of the Sundarbans, West Bengal. Environ and Ecol. 1985; 2: 243–248.

