# Supplementary figures and images for: Ranging, Activity and Habitat Use by Tigers in the Mangrove Forests of the Sundarban
Source: PLoS One. 2016 Apr 6;11(4):e0152119. doi: 10.1371/journal.pone.0152119 (PMC4822765; doi:10.1371/journal.pone.0152119)

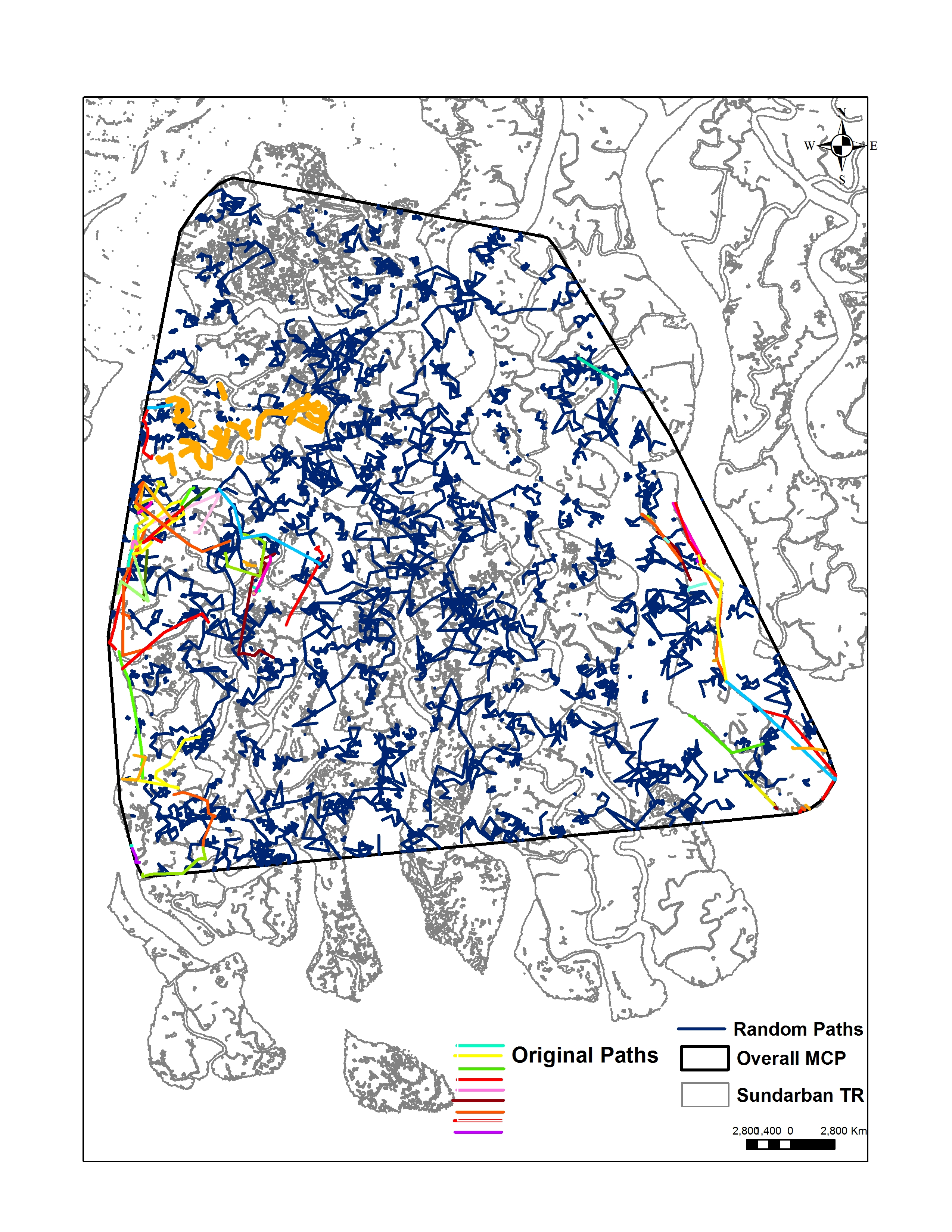

Supplement: S1 Fig — (TIFF) [file pone.0152119.s001.tiff]

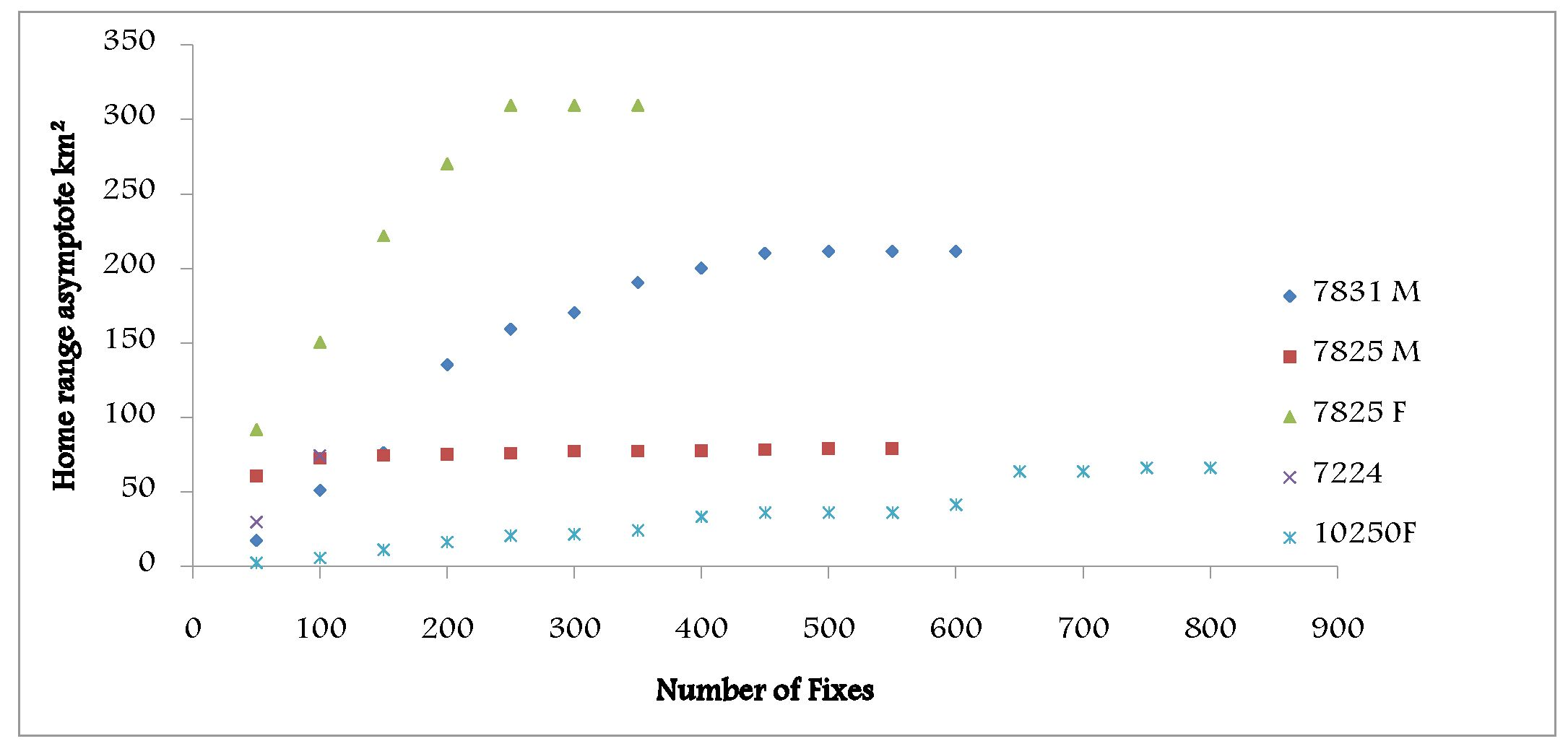

Supplement: S2 Fig — (TIFF) [file pone.0152119.s002.tiff]

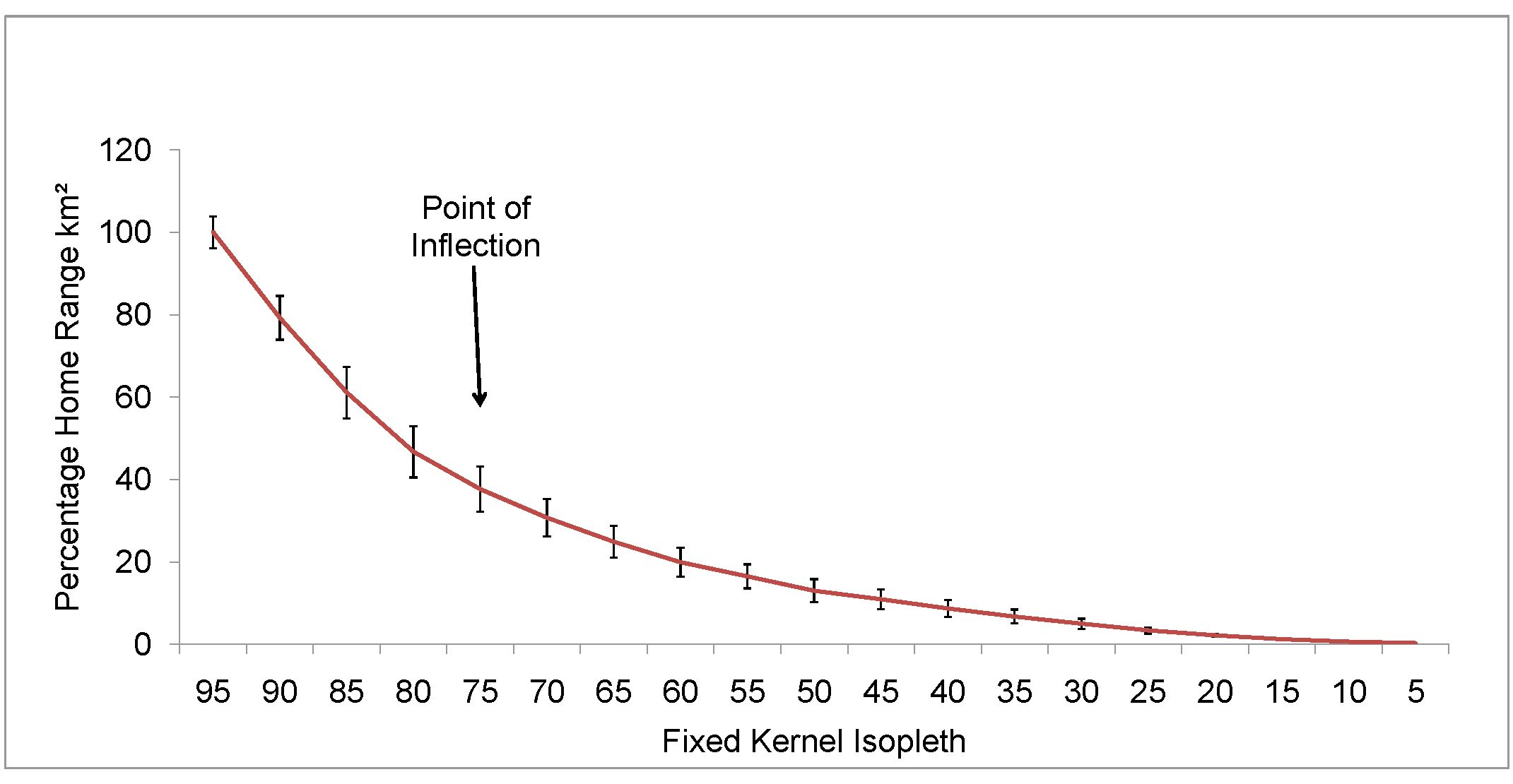

Supplement: S3 Fig — The point of inflection of the curve depicts the isopleth that best describes the core area of the home range, Error bars are standard errors. (TIFF) [file pone.0152119.s003.tiff]

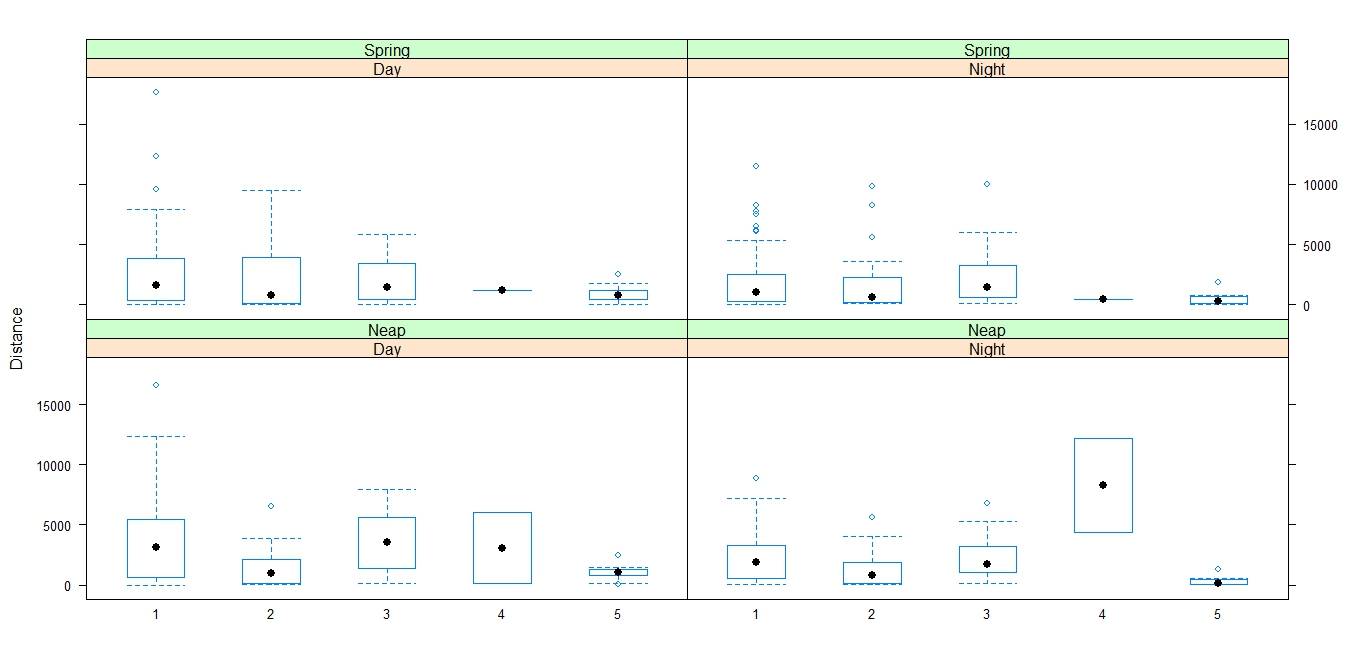

Supplement: S4 Fig — (TIFF) [file pone.0152119.s004.tiff]

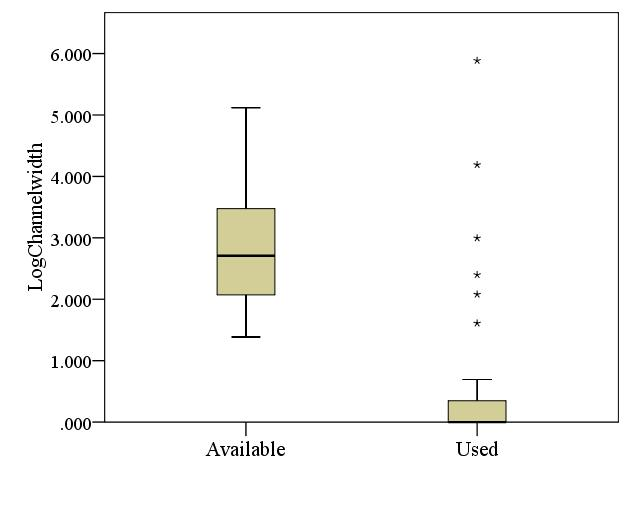

Supplement: S5 Fig — The data has been presented on a log scale. The bottom of the box indicates the 25th percentile. The top of the box represents the 75th percentile. The points outside the box are outliers. The asterisks or stars are extreme outliers. These represent cases/rows that have values more than three times the height of the boxes. (TIFF) [file pone.0152119.s005.tiff]

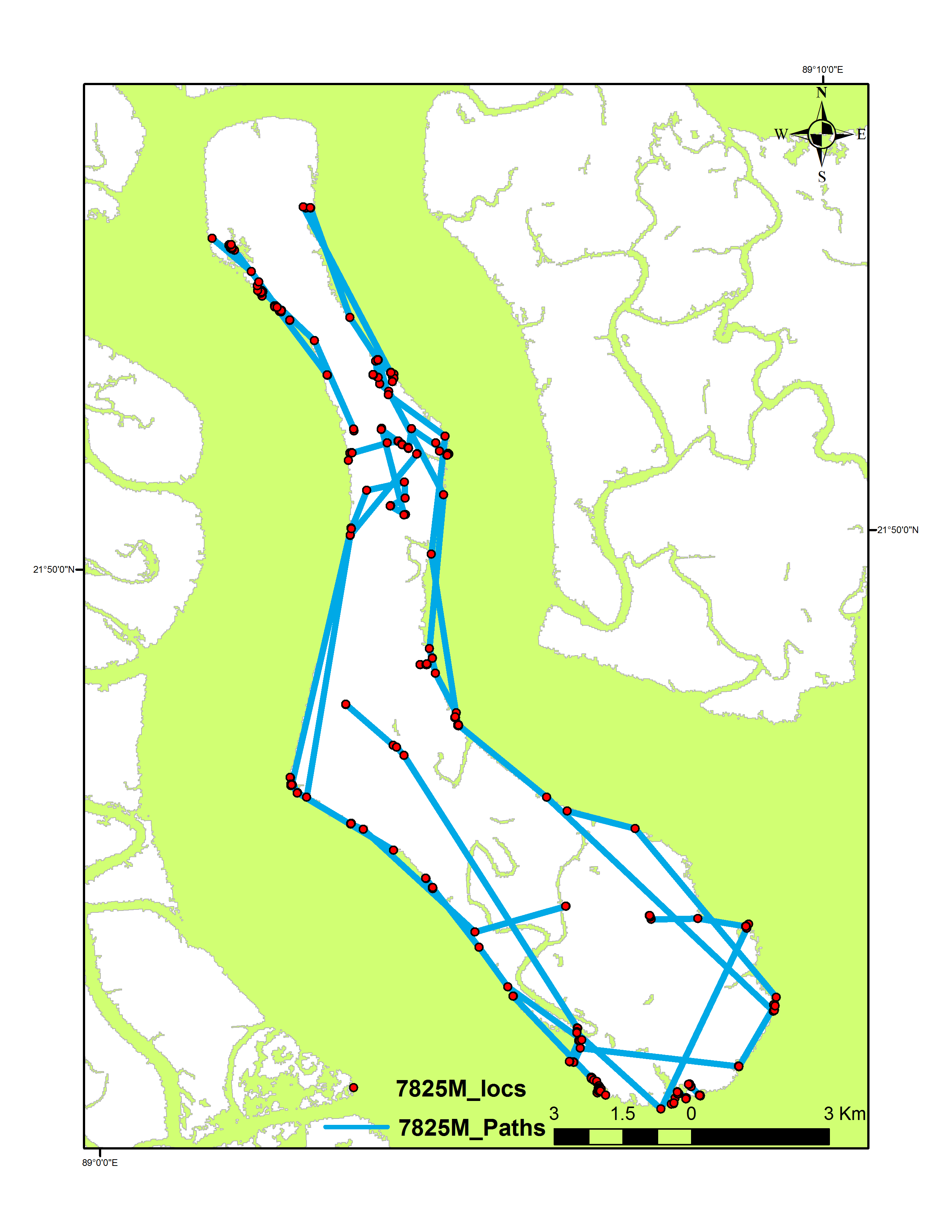

Supplement: S6 Fig — The path highlights the patrolling of shores of wide water channels by the tiger on Talpatti Island of Bangladesh. (TIFF) [file pone.0152119.s006.tiff]
